# Use of active learning classrooms in health professional education: A scoping review

**DOI:** 10.1016/j.ijnsa.2023.100167

**Published:** 2023-11-16

**Authors:** Hanne Maria Bingen, Hege Ingeborg Aamlid, Brit Marie Hovland, Andréa Aparecida Goncalves Nes, Marie Hamilton Larsen, Karoline Skedsmo, Eline Kaupang Petersen, Simen A. Steindal

**Affiliations:** a Lovisenberg Diaconal University College, Oslo, Norway; b VID Specialized University, Oslo, Norway

**Keywords:** Education, Learning, Learning environment, Medical education, Nursing, Teaching methods, Review

## Abstract

**Background:**

Active learning approaches, such as the use of active learning classrooms, can be an important measure to prepare health professional students for work-life. In addition to teaching approaches, the design of the classroom or learning spaces could facilitate learning. Although active learning classrooms are used in health professional education, no previous scoping review has investigated their use and associated outcomes.

**Objective:**

A scoping review was conducted to provide an overview of existing studies on the use of active learning classrooms in health professionals’ education.

**Design:**

Scoping review using the framework of Arksey and O'Malley.

**Methods:**

A systematic search was performed in ERIC, CINAHL, PsycINFO, Ovid MEDLINE, and Ovid EMBASE for papers published between January 2012 and January 2023. Hand searches of the reference lists of the included studies were also conducted. Inclusion criteria were as follows: studies using qualitative, quantitative, or mixed methods; studies including undergraduate, graduate, or postgraduate health professional students or teachers; studies that investigated students’ and teachers’ use of active learning classrooms or similar concepts in higher education; and studies published in English, Swedish, Danish, Norwegian, Spanish, or Portuguese in peer-reviewed journals. Pairs of authors independently assessed the eligibility of the studies and extracted the data, which were thematically grouped. The scoping review protocol was published with the Open Science Framework prior to the study selection process.

**Results:**

The review included 11 papers from 10 studies. Most studies were published between 2018 and 2022, exploring students’ perspectives. Three thematic groups were identified, suggesting that active learning classrooms (1) enhance a positive atmosphere and engagement but can be challenging; (2) facilitate students’ interactions; and (3) have the potential to stimulate active learning and co-construction.

**Conclusions:**

The use of active learning classrooms in health professional education can facilitate interactions among students, between students and teachers, and between students and class content. However, the design of active learning classrooms can both enhance and challenge students’ engagement and active participation. Teachers do not necessarily change their teaching approaches when compared to teaching in a traditional classroom. Future research should explore how to facilitate teachers’ use of the opportunities in active learning classrooms in health professional education and students’ learning outcomes, as well as the effect of high-technology active learning classrooms versus low-technology active learning classrooms on the intended learning outcome.

**Tweetable abstract:**

The use of active learning classrooms can facilitate active learning, but teachers do not necessarily change their teaching methods.

## What is already known

• Active learning approaches are recommended to prepare health professional students for their work-life.
• A transformation to more active learning to train health professional students has been recommended.
• A connection between teaching approaches and the design of the classroom has been previously described; however, no scoping review has investigated the use of active learning classrooms in health professional education.

## What this paper adds

• The features of active learning classrooms can provide opportunities for teachers to offer active learning in health professional education.
• The use of active learning classrooms has the potential to enhance students’ engagement, interactions, and active learning.
• Additional research on how teachers can use opportunities in active learning classrooms to facilitate students’ learning outcomes is necessary.

## 1 Background

Health professionals maintain and promote human health and prevent physical and mental impairments (WHO, 2013). An aging population leads to an increase in the prevalence of chronic illness and multimorbidity, demanding complex treatment options. These demands create new challenges for the healthcare system as a provider of healthcare (Sleeman et al., 2019; Zazzara et al., 2019). Educational institutions should prepare health professional students to meet these new challenges that call for a patient–provider partnership and involve collaborative care (Allegrante, 2018; Willman et al., 2021). Hence, the training of health professional students should apply active learning approaches (Bogetz et al., 2015). Health professional education encompasses the preparation of graduates for the professions of medicine, nursing, dentistry, pharmacy, occupational therapy, physical therapy, clinical psychology, and speech-language pathology (WHO, 2013).

Active learning strategies may facilitate learning in health professional education (Waltz et al., 2014), and active learning and student-centered approaches are recommended effective teaching methods to prepare health professional students for work-life (Bogetz et al., 2015). The core elements of *active learning* are student activity and engagement in the learning process, which are often contrasted with traditional lectures in which students more passively receive information (Prince, 2004). In student-centered environments, attention is “on what the students are doing; hence, it is the student's behavior that is the significant determinant of what is learned” (Michael, 2006, p. 160). Active learning can allow students to participate in learning activities, take responsibility for their learning, and establish connections between ideas by analyzing, evaluating, and creating (Gogus, 2012).

Systematic reviews have shown that the *flipped classroom* facilitates active learning and supports health professional students’ learning (Barranquero-Herbosa et al., 2022; Silva et al., 2021) and is one approach to preparing students for a complex healthcare practice environment (Barbour & Schuessler, 2019). This approach emphasizes more time in class for interactive learning activities (Bishop & Verleger, 2013) and may positively affect student performance and facilitate the acquisition of cognitive skills, such as applying, analyzing, evaluating, and creating (Chung et al., 2019; Leatherman & Cleveland, 2019; Strelan et al., 2020). A review has shown that the flipped classroom may support students’ learning because of learning activities in class that require higher-order cognitive processes. Activities that positively affect student learning include, for example, response system questions with group discussions and teacher's explanations, pair-and-share activities, and small group discussions (DeLozier & Rhodes, 2017). Another example is student presentations, in which students create, present, and teach content to their peers (DeLozier & Rhodes, 2017).

Studies have indicated that teaching approaches and various pedagogies to engage students can be affected by the design of the classroom and the opportunities to facilitate dialog and collaboration (Siegel & Claydon, 2016; Wilson & Randall, 2012), and higher education has invested in new kinds of learning spaces to support a broad range of pedagogical approaches (Ellis & Goodyear, 2016). Traditional classrooms have been redesigned to better utilize students’ limited time in class. One example is the student-centered active learning environment with upside-down pedagogies, which includes redesigned instructional space and reformed pedagogy (Beichner, 2014). Such spaces are an example of an active learning classroom that could facilitate active learning and in-class learning activities within the flipped classroom approach.

The active learning classroom has no universal definition but has typical features to support students’ group work and opportunities to share these works with the entire class (Baepler et al., 2016). According to Talbert and Mor-Avi (2019), descriptions of such spaces have several common characteristics. Based on this view, we describe active learning classrooms as formal spaces where students convene for educational activities and include design attributes specifically intended to promote additional active learning, such as movable furniture. The seating places students in small groups with a writing surface per group, as active learning classrooms have no defined front of the room. Instead, the teacher has a station that is either movable or located in an inconspicuous location. Further, active learning classrooms have access to digital and analog tools for learning, including projectors, tablets or laptop computers, whiteboards, and classroom response systems.

In a literature review of research on active learning classrooms, since the introduction of the student-centered active learning environment with upside-down pedagogies until 2017, researchers have examined the effects of active learning classrooms (Talbert & Mor-Avi, 2019). The review included studies from different disciplines in higher education but none from health professional education courses. A comparison of traditional lectures with active learning classroom lectures revealed that students in active learning classrooms either performed better or no significant differences were observed in measurable learning outcomes such as exam, course grades, or reduction in the failure rates (Talbert & Mor-Avi, 2019). The improvement was most pronounced among low-achieving students, and a study included in their review showed that “the bottom 25 % of students in the [active] section scored significantly higher than the bottom 25 % of students in the traditional section on the last three exams” (Talbert & Mor-Avi, 2019, p. 9). Compared to traditional classrooms, students reported a preference for learning in active learning classrooms, and a study showed that “78 % of students preferred the flexible design over traditional fixed design” (Talbert & Mor-Avi, 2019, p. 13). Students described increased motivation and willingness to participate actively, worked beyond their comfort zone, and experienced increased interaction and enhanced relationships with peers and teachers (Talbert & Mor-Avi, 2019). Teachers tended to change their perceptions of their teaching roles and practices and used active learning approaches more frequently, and they tended to integrate the affordances of active learning classrooms into their teaching, spending less time lecturing and more time moving around the classroom, engaging in discussions with students, and facilitating group activities (Talbert & Mor-Avi, 2019). A scoping review of the connection between learning and physical learning spaces and how it could inform health professional education indicates that most research studies have focused on how the use of technology can enable active learning (Nordquist, 2016). One of the studies included in the review was on a health professions course that used an active learning classroom (Nordquist, 2016).

Active learning approaches, such as active learning classrooms, could be an important measure to prepare health professional students to collaborate with other healthcare professionals and to provide care and treatment in complex patient situations. However, students’ perceptions of learning experiences and the level of positive effects of active learning classrooms seem to vary across academic disciplines (Chiu et al., 2022), and studies from other disciplines in higher education may not be transferable to health professional education. While there have been studies exploring the integration of active classrooms into healthcare professional education, a comprehensive search of the literature did not yield scoping reviews that specifically delve into the utilization of active learning classrooms in the context of health professional education. Therefore, we aimed to conduct a scoping review to summarize the range of studies and existing findings, identify the gaps in this field of research, and investigate the potential for a systematic review (Arksey & O'Malley, 2005; Pollock et al., 2021). The aim of this scoping review was to provide an overview of published studies on the use of active learning classrooms in health professional education. We asked the following research question: What is known from existing studies about the use of active learning classrooms in health professional education?

## 2 Methods

The scoping review employed the methodological framework described by Arksey and O'Malley (2005) and updated the methodological guidance for scoping reviews (Peters et al., 2020; Pollock et al., 2021). The scoping review protocol was published with the Open Science Framework (https://osf.io/ctewz/). Changes in the protocol are reported in Appendix 1.

### 2.1 Eligibility criteria

The eligibility criteria are described in Table 1 using the population, concept, and context framework (Peters et al., 2021).

**Table 1:** Eligibility criteria.

Criterion	Inclusion	Exclusion
Population	Studies including undergraduate, graduate, or postgraduate health professional students or teachers. This includes students and teachers from medicine, nursing, dentistry, pharmacy, occupational therapy, physical therapy, clinical psychology, and speech-language pathology, and from courses like public health, health sciences, biomedicine, biostatistics, and data science.	Studies including other students or teachers than health professional students or teachers
Concept	Students’ and teachers’ use of active learning classroom or similar concepts, such as active learning environment, active learning space, active learning center, or student-centered active learning environment with upside-down pedagogies to facilitate learning Characteristics of active learning classrooms: 1. Formal spaces in which students convene for educational activities 2. Design attributes specifically intended to promote active learning 3. No defined front of the classroom 4. Access to digital and analog tools for learning	Campus design, architecture, space design, skill or simulation lab, augmented reality, virtual reality, virtual rooms, and escape rooms
Context	Higher education Health professional education Educational programs and courses in clinical practice Use of active learning classrooms regardless of setting (also used in education in the clinical setting)	Primary or secondary education Education other than health professional education, courses, or clinical settings
Outcome	Subjective and/or objective learning outcomes	
Types of sources of evidence	Qualitative, quantitative, or mixed methods studies on the concept published in peer-reviewed journals	Master's or PhD theses, all types of reviews, conference abstracts, conference proceedings, protocols, editorials, letters, comments, books, book chapters, or guidelines

### 2.2 Information sources

A systematic search was performed on May 3, 2022 in ERIC, CINAHL, PsycINFO, Ovid MEDLINE, and Ovid EMBASE to identify relevant papers published between January 1, 2012, and May 3, 2022. The systematic search was updated on January 23, 2023. This period was selected based on a previous review study that found that most active learning classroom studies were published between 2012 and 2016 (Talbert & Mor-Avi, 2019), and the first study regarding health professional education published in 2013 (Nordquist, 2016). The reference list of the included papers and related reviews identified in the search was manually searched to identify relevant papers.

### 2.3 Search

Based on an initial search, an experienced research librarian collaborated with the first and last authors to build the search strategy in Ovid Medline using text words to denote various types of active learning classrooms or similar environments. Medical subject headings were not used, as no suitable terms were available to describe these environments. The strategy was piloted by the first and last authors. The search strategy was then adopted for other databases. A second research librarian reviewed the search strategy for all the databases using the Peer Review of Electronic Search Strategies checklist (McGowan et al., 2016). The final search strategies are shown in Appendix 2.

The database searches were limited to papers published in English, Swedish, Danish, Norwegian, Spanish, or Portuguese, as the research team understands these languages.

### 2.4 Selection of sources of evidence

The librarian transferred the identified publications to EndNote and used the de-duplication method to remove duplicates (Bramer et al., 2016). To ensure that the eligibility criteria were consistent across the pairs of reviewers, the authors discussed the meaning of the inclusion and exclusion criteria (Pollock et al., 2021). The process for selecting the sources of evidence was conducted in two steps to screen and assess whether the publications satisfied the eligibility criteria: (1) screening of titles and abstracts using the web application Rayyan (Ouzzani et al., 2016) to facilitate blinding and (2) screening of full-text publications. In both steps, the pairs of authors independently assessed whether the publications met the eligibility criteria. When disagreement or uncertainty occurred, the pairs discussed whether a publication met the inclusion criteria. When the uncertainty about whether a publication should be included remained, the first author was consulted, and the final decision was based on a consensus between the pair and the first author.

### 2.5 Data charting process

We developed a standardized data charting form to collect the following information from the included papers: author, year, and country; design; aim; sample (characteristics, sample size); description of the active learning classroom, including use of technology in active learning classroom (design attributes and digital and analog tools); learning activities facilitated in active learning classroom; and didactics; and findings related to the research question. The first and last authors piloted the data charting form by extracting data from two papers to ensure consistency and that all relevant data were captured appropriately (Peters et al., 2020; Pollock et al., 2021). Based on the piloting, the data charting form was adjusted. The pairs of authors extracted the data. One author extracted the data, while the other checked the accuracy. In cases of disagreement, the first and second authors independently extracted the data and made a final decision.

### 2.6 Synthesis of results

A scoping review provides an overview and summary of the results but does not intend to synthesize the results to inform practice or policymakers. The analysis of the results from the results section of the included papers is normally descriptive (Pollock et al., 2021). However, a scoping review requires an analytical framework; thus, the data extracted in this study were thematically grouped (Arksey & O'Malley, 2005). The first, second, and last authors used a qualitative approach to thematically group the data. We transformed the numerical data presented in tables and figures into a qualitative format (Lundereng et al., 2023). First, the extracted data were read several times to produce an overview. Second, guided by the research question, the data were read to identify patterns of differences and similarities across the included papers. These patterns were organized into thematic groups, and the analysis was on a descriptive (manifest) level. This approach to thematic grouping has previously been used in scoping reviews (Nes et al., 2021; Steindal et al., 2020). All authors agreed on the final thematic groups to facilitate trustworthiness and intersubjectivity.

To identify the research gaps, we presented the characteristics of the included studies in Appendix 3. A frequency table was used to illustrate the papers that were included in the groups (see Table 2) (Nes et al., 2021; Steindal et al., 2020).

**Table 2:** Papers included in thematic groups.

Thematic groups	Study		Number of papers
	Students’ perspectives	Teachers’ perspectives	
Enhance positive atmosphere and engagement but can be challenging	Donkin & Kynn (2021), Gordy et al. (2018), Gordy et al. (2019), Gordy et al. (2020), Marchiori & McLean (2022), Metzger & Langley (2020), Seet et al., (2022)	Gordy et al., (2018), Gordy et al., (2019)	7
Facilitate student interactions	Donkin & Kynn (2021), Bruner et al. (2022), Gordy et al. (2018), Gordy et al. (2019), Gordy et al. (2020), Marchiori & McLean (2022)	Gordy et al., (2018), Gordy et al., (2019)	6
Potential to stimulate active learning and co-construction	Donkin & Kynn (2021), Bruner et al. (2022), Gordy et al. (2018), Gordy et al. (2019), Gordy et al. (2020), Lee et al. (2018), Marchiori & McLean (2022)	Basdogan & Morrone (2021), Beery et al. (2013), Gordy et al. (2018), Gordy et al., (2019)	9

## 3 Results

### 3.1 Selection of sources of evidence

**Fig. 1:**
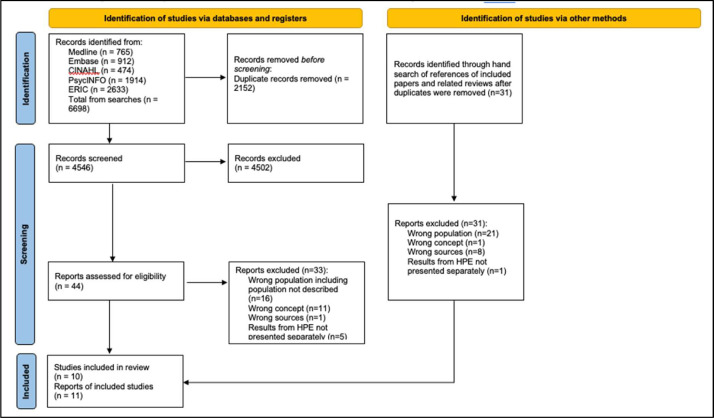
Summary of the selection of sources.

The database searches yielded 4546 publications after duplicates were removed. The titles and abstracts were screened, and based on the inclusion and exclusion criteria, the full text of 44 publications was read, 33 publications were excluded, and 11 papers were included in the review. Hand searches of the included papers yielded no additional papers. The reasons for the exclusion of full-text papers are shown in Fig. 1.

### 3.2 Description of included studies

A total of 11 papers from 10 studies published in 2013 (*n* = 1) and between 2018 and 2022 (*n* = 10) were included. Two of the papers were from the same study—a qualitative study (Gordy et al., 2018) and a mixed methods study (Gordy et al., 2019). The studies were conducted in the United States (*n* = 7), Canada (*n* = 1), Australia (*n* = 1), and Singapore (*n* = 1). Six papers used mixed methods, one used a qualitative interpretive design, one used an experimental design, two used a quasi-experimental design, and one used a cross-sectional design.

Nine papers included students in biomedicine (*n* = 1), dental hygiene (*n* = 3), speech-language pathology (*n* = 1), physical therapy (*n* = 1), occupational therapy (*n* = 1), biostatistics and data science (*n* = 1), public health (*n* = 1), medicine (*n* = 2), and health sciences (*n* = 1). The sample size of the included studies ranged from 32 to 193 health professional education students. The majority of the students were females in four papers and males in one paper, with almost an equal number of females and males in one paper, whereas three papers did not report gender. The students’ ages ranged from 18 to 25 years in four papers, whereas age was not reported in five papers. Four papers included teachers in public health (*n* = 1), nursing (*n* = 1), and dental hygiene (*n* = 2). The sample size of the included studies ranged from 1 to 8 health professionals’ education teachers.

Papers investigated the influence of active learning classrooms on teaching and learning and the support of active learning (Basdogan & Morrone, 2021; Gordy et al., 2018; Gordy et al., 2019; Lee et al., 2018). Papers also investigated teachers’ and students’ behaviors, students’ active engagements and interactions taking place and how these interactions affect learning (Beery et al., 2013; Gordy et al., 2020; Metzger & Langley, 2020). There were also investigations of in-class activities within the flipped classroom approach, the effects of classroom architecture and pedagogical design on student achievement (Bruner et al., 2022), and the impact of an active learning classroom on the development of communication skills and the quality of peer-to-peer interactions (Marchiori & McLean, 2022). Furthermore, the impact of active learning classroom on facilitating the teaching approach of team-based learning and the effect of seating on engagement were investigated (Donkin & Kynn, 2021; Seet et al., 2022).

Ten papers described the physical attributes of an active learning classroom. All rooms were equipped with movable chairs and group tables. Some rooms also had swivel chairs with built-in work surfaces and storage for personal belongings (Gordy et al., 2020; Gordy et al., 2018; Gordy et al., 2019) or sofa seats with coffee tables (Basdogan & Morrone, 2021). The tables seated 3–8 students (Bruner et al., 2022; Donkin & Kynn, 2021; Lee et al., 2018; Seet et al., 2022), and the capacity of the rooms was 49–96 students (Basdogan & Morrone, 2021; Donkin & Kynn, 2021; Lee et al., 2018). The teacher's stations were in the center of the room (Lee et al., 2018; Marchiori & McLean, 2022), in the front corner (Lee et al., 2018), or in front, and the students were seated circularly (Seet et al., 2022).

The students used technology, including portable, wall-mounted, digital, or interactive whiteboards (Basdogan & Morrone, 2021; Beery et al., 2013; Bruner et al., 2022; Gordy et al., 2020; Gordy et al., 2018; Gordy et al., 2019; Lee et al., 2018; Marchiori & McLean, 2022), laptops, tablets, or dedicated networked computers (Basdogan & Morrone, 2021; Beery et al., 2013; Donkin & Kynn, 2021), microphones (Bruner et al., 2022; Lee et al., 2018; Seet et al., 2022), acoustic amplifiers (Bruner et al., 2022), wireless keyboards and mice (Beery et al., 2013), and power outlets and hook-ups for personal devices (Gordy et al., 2020; Gordy et al., 2018; Gordy et al., 2019; Lee et al., 2018). The technology also included document cameras (Lee et al., 2018), webcams, a copy camera to digitally capture whiteboards, and the opportunity for students and the teacher to retrieve their digital copies from an internal website (Beery et al., 2013). The technology the teachers used included whiteboards (Basdogan & Morrone, 2021; Beery et al., 2013), computers (Basdogan & Morrone, 2021; Lee et al., 2018), control panels (Lee et al., 2018), microphones (Bruner et al., 2022; Lee et al., 2018), acoustic amplification (Bruner et al., 2022), and projector and screen (Basdogan & Morrone, 2021).

The room's projectors and screens varied among the studies and could be placed around the periphery of the room (Seet et al., 2022), on interconnected screens on the walls (Gordy et al., 2020, 2018, 2019; Lee et al., 2018), at the tables (Gordy et al., 2020; 2019; Lee et al., 2018; Marchiori & McLean, 2022), or near each table connected to student's laptop (Bruner et al., 2022). One room had a video wall (Lee et al., 2018). In one room, each group had a screen that could display the teacher's secondary monitor or the group's dedicated computer (Beery et al., 2013). In another room, each group had a touchscreen computer linked to a lectern screen and could use the central display monitor projected onto the lectern screen to view and comment on the central display that contained each group's activities (Donkin & Kynn, 2021). In addition, the room's other equipment included color-coded group icons, synchronous collaboration software to involve distance-learning students, duplicated primary displays (Beery et al., 2013), and clickers (Basdogan & Morrone, 2021).

Learning activities described in the papers included different modes and approaches, for example, lectures with PowerPoint presentations and videos (Basdogan & Morrone, 2021; Beery et al., 2013; Lee et al., 2018), which could be one-way or interactive, including dialogues and the use of clickers (Basdogan & Morrone, 2021; Beery et al., 2013; Lee et al., 2018). The teachers lectured before or after group activities and shifted from one teaching mode to another, offering different classroom activities (Gordy et al., 2019; Lee et al., 2018). The activities could be individual work, followed by sharing, presenting, and discussing (Gordy et al., 2018; Gordy et al., 2019; Lee et al., 2018). Other activities included group work, including group presentations and discussions, role play and peer teaching, and class-wide discussion (Gordy et al., 2020; Gordy et al., 2018; Gordy et al., 2019; Lee et al., 2018). Furthermore, using a flipped classroom approach, activities in class included activities based on students’ baseline knowledge (Marchiori & McLean, 2022) and individual quizzes and problem-based learning assignments in teams of students (Bruner et al., 2022). Using a team-based learning approach, learning activities included feedback, individual assessment, peer review through group presentations (Donkin & Kynn, 2021), and readiness assurances and application exercise (Seet et al., 2022).

Didactic or teaching approaches described in the papers included flipped classroom (Bruner et al., 2022; Marchiori & McLean, 2022), team-based learning (Donkin & Kynn, 2021; Seet et al., 2022), and direct instruction, monitoring, and consulting (Basdogan & Morrone, 2021). The rest of the papers did not report on the teaching approach used. The characteristics of the included studies are shown in Appendix 3.

### 3.3 Thematic groups

To answer the research question regarding what is known from existing studies about the use of active learning classrooms in health professional education, the results are presented in three thematic groups; (a) enhance positive atmosphere and engagement but can be challenging, (b) facilitate students’ interactions, and c) have the potential to stimulate active learning and co-construction.

### 3.4 Enhance positive atmosphere and engagement but can be challenging

In three papers, the atmosphere in the active learning classroom was different from that in the traditional classroom (Gordy et al., 2020; Gordy et al., 2018; Gordy et al., 2019). The physical features of the active learning classroom made students feel freer, and they experienced the learning environment as significantly more welcoming, comfortable, and relaxed compared with the traditional classroom. The learning space was described as “non-hierarchical,” which facilitated a “positive psychological climate conducive to learning,” and the students described it as “a creative vibe in the room.” The learning environment “promoted reciprocated peer relations,” and students interacted and socialized more easily with their peers (Gordy et al., 2020; Gordy et al., 2018; Gordy et al., 2019). During in-class activities within the flipped classroom, some students described the active learning classroom as “more comfortable” than the traditional classroom, while others stated that the traditional classroom was “less awkward and more comfortable” (Marchiori & McLean, 2022).

Active learning classroom design could promote student engagement, and the teacher described the students as being more committed to staying in the room than in traditional classrooms (Gordy et al., 2018). Students described that staying focused and engaged, paying attention, and participating actively was easy and that they were more excited about attending the active learning classroom than a traditional classroom (Gordy et al., 2020, 2018, 2019). Students showed the following engagement behaviors: listening, discussing, problem-solving, writing, and reading (Metzger & Langley, 2020). Even though the most observed student engagement behavior was listening, approximately all observed student time was accounted for by listening, discussing, and problem-solving (Metzger & Langley, 2020).

Students’ cognitive engagement could be promoted in an active learning classroom during team-based learning (Seet et al., 2022). Even though students preferred sitting with their front facing the teacher, seating orientation did not significantly affect their engagement. The seating distance to the teacher did not significantly affect second-year students; however, the engagement of first-year students who moved farther away from the teacher decreased significantly (Seet et al., 2022). In one study, 78 % of the students reported that the use of modern technology was a motivator to learn, and 30 % reported that the technology in the room was useful and enjoyable for the learner during team-based learning (Donkin & Kynn, 2021). Furthermore, students in this study described that the environment in the active learning classroom made concentrating and engaging easy and teaching easy to follow.

Other students described that the design of the active learning classroom and the use of technology could cause “distraction” (Gordy et al., 2019). Donkin and Kynn (2021) found that 7.5 % of students reported that technology created problems or wasted time.

### 3.5 Facilitate students’ interactions

In three papers, both teachers and students described that the active learning classroom's non-hierarchical design enhanced student–student and student–teacher classroom interactions (Gordy et al., 2020, 2018, 2019). The freedom to move around could facilitate interactions with peers and interactive learning activities (Gordy et al., 2018). Gordy et al., (2020) found that “the increased interactions with heterogeneous groups of peers promoted overall peer relations in class.”

Students experienced that the design of the active learning classroom better supported student–student interactions and preferred active learning classrooms over traditional classrooms during group work. The design significantly made the execution of group activities easy, enhanced group work efficiency, and allowed easy and effective collaboration (Gordy et al., 2020, 2018, 2019). In one study, 22.5 % of students reported that the collaboration was good for group work and/or improved the group work experience (Donkin & Kynn, 2021). Furthermore, students experienced that the active learning classroom had a significantly higher impact than the traditional classroom on group work, collaboration, and student–student interactions during in-class activities within the flipped classroom approach (Marchiori & McLean, 2022). Therefore, they preferred an active learning classroom to support communication skill development. Students scored the active learning classroom significantly higher than the traditional classroom, especially regarding feelings about the physical classroom during in-class activities (Bruner et al., 2022).

Most students “believed their peers in their team were knowledgeable, enthusiastic to learn, reliable, and well-prepared” during team-based learning (Donkin & Kynn, 2021) and significantly experienced that peer collaboration and interaction in the group increased learning accountability, and students described a reduced occurrence of social loafing (Gordy et al., 2020). Students significantly experienced that everyone in the group participated and expressed higher peer motivation to contribute to the group work because working with a group “forced you to keep up” (Gordy et al., 2020).

### 3.6 Have the potential to stimulate active learning and co-construction

The possibility of tailoring and stimulating teaching in an active learning classroom was described. A teacher described a need to change the teaching approach in the active learning classroom due to the challenge of making visual contact with all students, and students significantly experienced that the teachers offered more time for discussions and group work in the active learning classroom than in the traditional classroom (Gordy et al., 2019). In the active learning classroom, students described more attention from the teacher and confirmation of their understanding, more interactions with the class content and material being taught, and more work-related scenarios, as well as interactions with real-life examples (Donkin & Kynn, 2021; Gordy et al., 2018; Gordy et al., 2019).

In the active learning classroom, teachers seemed to offer learning activities in which students had to participate actively, and the room design facilitated collaboration and interactive activities (Gordy et al., 2018; Gordy et al., 2019). The teacher easily took control and designated tasks—for certain students or all—based on the activities (Gordy et al., 2019). This environment could foster creativity and provide teachers with the opportunity to involve students in higher-order thinking (Gordy et al., 2018). Students described more active learning and hands-on activities in the active learning classroom than in the traditional classroom (Gordy et al., 2020; Gordy et al., 2019). Hands-on group work and being in a small group could facilitate students’ mutual learning and foster idea generation and co-construction of knowledge or the ability to build upon each other's expertise, as well as experiences of being more creative and stimulating their critical thinking (Gordy et al., 2020). Furthermore, the atmosphere and sitting in a group could encourage students to speak in class (Gordy et al., 2020). Teachers in active learning classrooms could facilitate class-wide discussions using video walls to present student work (Lee et al., 2018). The active learning classroom could provide an interactive learning style, and students described an enhanced ability to learn individually and within a group and engage with the class in constructive conversations (Donkin & Kynn, 2021).

Regarding teachers’ most prevalent pedagogic approach, a teacher's behavior in the active learning classroom was characterized by direct instruction and monitoring, roaming around the room, consulting interactive dialogs with the students, and using clickers (Basdogan & Morrone, 2021). Teachers using an active learning approach seemed to behave similarly in active learning and traditional classrooms, whereas teachers using a passive learning approach seemed to use the same approach in both rooms (Beery et al., 2013). Similarly, no significant differences were found between the interactions in the active learning classroom and the traditional classroom (Beery et al., 2013).

Within a flipped classroom approach, students scored the active learning classroom as similar to the traditional classroom in terms of pedagogy and their course experiences in the classroom (Bruner et al., 2022). Neither the students’ perceptions of the classroom spaces nor the spaces themselves significantly predicted the final course grade (Bruner et al., 2022). However, students who scored their course experiences and employed pedagogy higher also performed better. Working with peers and teaching and learning with peers were most positively related to grade, whereas displaying student work in front of the class was most negatively related to grade (Bruner et al., 2022). Nevertheless, the room had no quantifiable effect on students’ communication apprehension, and they seemed to behave similarly in the active learning classroom and traditional classroom when performing in-class activities; most students preferred the active learning classroom to support communication skill development (Marchiori & McLean, 2022).

Regarding students’ achievement during team-based learning, students in active learning classrooms performed significantly better than students in traditional classrooms on their final examinations (Donkin & Kynn, 2021). However, concerning their learning experiences, approximately 3 % of the students reported that using technology and working in a group inhibited learning (Donkin & Kynn, 2021).

## 4 Discussion

Our scoping review aimed to provide an overview of peer-reviewed studies on the use of active learning classrooms in health professional education. Our findings suggest that teachers’ and students’ freedom to move around the classroom, as well as students’ common work surfaces, can facilitate a student-centered environment and affect students’ active participation. The atmosphere and design of active learning classrooms seemed to support a learning environment that facilitated interactions among students and between students and teachers. Furthermore, both students and teachers reported more active learning in active learning classrooms than in traditional classrooms; however, whether the teaching approach changed correspondingly remain uncertain.

Our findings highlight the opportunity for teachers and students to move around during class and the students’ use of artifacts, such as portable or digital whiteboards and screens, which made seeing, participating, and sharing easy. This finding is consistent with a previous review (Talbert & Mor-Avi, 2019). However, one of the included studies found that displaying student work on screens was negatively related to the final course grade (Bruner et al., 2022). Fukuzawa and Cahn (2019) found that the visibility of the groups’ discussion boards can hinder interactions between students, since they want to prevent other groups from “stealing” their ideas and avoid feeling less productive when viewing other groups’ discussion boards. Recent studies also show that using digital tools in class to visualize students’ understandings can encourage them to participate; however, it can also hinder those with low self-efficacy from participating actively in learning activities (Bingen et al., 2020; Bingen et al., 2019).

Our findings also suggest that the opportunity for students to move around can impact the atmosphere in active learning classrooms, creating an informal and relaxed learning environment that support experiences of confidence. Compared with traditional classrooms, the design and use of active learning classrooms seem to underpin the aspect of active learning environments as student-centered (Michael, 2006), placing the students in the center of the room. Active learning classrooms underpin a change in the teacher's role, from “a sage” to “a guide” (McLean & Attardi, 2018), and the distribution of power due to a “non-hierarchical” learning environment.

Our findings show that a positive atmosphere and opportunity for socializing can facilitate a learning environment with engagement, interactions, and effective collaboration in active learning classrooms (Donkin & Kynn, 2021; Gordy et al., 2020; Gordy et al., 2018; Gordy et al., 2019; Marchiori & McLean, 2022; Metzger & Langley, 2020). Even though active learning classrooms seemed to have a positive or no measurable effect on students’ achievement or learning outcomes in the studies reviewed, the findings indicate that students preferred them over traditional classrooms due to the support of interactions (Bruner et al., 2022; Donkin & Kynn, 2021; Marchiori & McLean, 2022). Talbert and Mor-Avi (2019) indicated that the term *engagement* is poorly operationalized and suggested using a framework that includes affective, behavioral, and cognitive engagement when investigating active learning classrooms. One study in our scoping review investigated cognitive engagement (Seet et al., 2022), while another investigated student engagement behavior (Metzger & Langley).

Our findings suggest that interactions in active learning classrooms are related to engagement and that enhanced engagement facilitates interactions. Even though the students seemed to interact and discuss more with their peers, the observed discussions might have been non-professional. However, according to two of the included papers (Gordy et al., 2020; Gordy et al., 2018), students seemed more committed to participating actively, and social loafing was reduced, as students experienced a sense of ownership over what their group did and contributed during group work. These findings are consistent with those of Bingen et al., (2020), who found that students experience more commitment to studying before small-group meetings than meetings in full class. Our findings also indicate that students in active learning classrooms experienced a decreased participation threshold due to a sense of belonging in the group they did not experience in traditional classrooms. Students experienced that peers met prepared for group work during team-based learning in the active learning classroom. Peers’ preparedness may influence students’ experiences of peers as capable others and may be important for peer interactions and facilitate collaboration (Bingen et al., 2020; Bingen et al., 2019). Our findings indicate that students paid more attention to teaching and experienced more attention and confirmation from teachers in active learning classrooms than in traditional classrooms (Donkin & Kynn, 2021; Gordy et al., 2020, 2018, 2019). Furthermore, sitting in a group can encourage students to speak in class in active learning classrooms. This finding can be due to experiencing support from peers in the group and being in a group with a higher collective self-efficacy (Bandura, 1997) than the self-efficacy one has on one´s own.

Our findings indicate that the learning activities offered in active learning classrooms can facilitate interactions with the content and material being taught and enhance hands-on, real-life, and work-related activities (Donkin & Kynn, 2021; Gordy et al., 2020, 2018, 2019). Active learning classrooms can provide opportunities to involve students in higher-order thinking and stimulate creativity, critical thinking, and learning through the co-construction of knowledge, and students’ experiences of the pedagogy employed could relate positively to the final grade. This finding is consistent with active learning strategies, which are the most used teaching and learning strategies in such rooms, and the most emphasized purpose of using educational technology is interaction (Carlos et al., 2023). Active learning allows students to participate in learning activities (Gogus, 2012). Active learning classrooms may facilitate such an approach by offering activities that can positively affect students’ learning within flipped classrooms (DeLozier & Rhodes, 2017). Our findings suggest that both students and teachers experienced more active learning in active learning classrooms than in traditional classrooms. However, observations showed that teachers behaved similarly in the active learning classroom and the traditional classroom and offered the same activities in the active learning classroom, similar to those provided in the traditional classroom. This finding indicates that teachers need time to change and adjust to their new roles in active learning classrooms. Whether active learning classroom design affects teachers’ teaching practices may depend on the teachers’ teaching philosophies and whether the teachers already facilitate active learning (Talbert & Mor-Avi, 2019).

Most of the studies in our review used students’ perspectives as assessment criteria to monitor the use of active learning classrooms. In student-centered environments, students’ behavior is important to what they learn (Michael, 2006). However, what the students learn is also interesting, and three studies in our review examined the students’ achievements (Bruner et al., 2022; Donkin & Kynn, 2021; Marchiori & McLean, 2022). Consequently, further research on students’ performance is necessary. According to a systematic review, well-designed formal learning spaces may support changes in pedagogy toward active learning methods that enable students to deeply understand a subject (Leijon et al., 2022). Our findings indicate a need for research on facilitating health professional education teachers’ use of opportunities in active learning classrooms.

Reviews of health professional education show that flipped classrooms’ effectiveness is positively affected by in-class activities based on active learning and interactions and negatively affected by students’ desire for passive explanations (Banks & Kay, 2022; Oudbier et al., 2022). Even though students perform better in active learning environments, they may think that they learn less than they do in passive environments because of the increased cognitive effort associated with active learning (Deslauriers et al., 2019). Furthermore, one study found no differences in students’ performances when the same in-class activities were offered in an active learning classroom and a traditional classroom (Stoltzfus & Libarkin, 2016). Only two studies in our review investigated the flipped classroom (Bruner et al., 2022; Marchiori & McLean, 2022); thus, further research on the impact of the active learning classroom regarding the facilitation of in-class activities, including students’ motivation to prepare during pre-class activities and experiences of learning, is necessary.

Based on the description of the design of active learning classrooms in the included studies, most of them were high-technology active learning classrooms. Nicol et al., (2017) found no differences in students’ performance in high-technology active learning classrooms versus low-technology active learning classrooms. Given the investment costs related to high-technology active learning classrooms, future studies should investigate whether the same is true for health professional education.

During the screening process, we excluded studies due to a lack of reporting descriptions of the participants’ affiliations with a specific education. Furthermore, included studies investigating active learning lacked a description of the room where the activities were offered and where the in-class activities were performed in flipped classrooms, and the used didactic, teaching design, and interventions were often not reported. Therefore, future research should describe the features of the active learning classroom and the didactic teaching design and interventions used.

Our review included most studies from the United States and none from Europe or Africa, which may be because more institutions in the United States have built active learning classrooms or performed research on such rooms or because studies from other continents are written in languages that we excluded.

## 5 Limitations

We conducted a systematic search to identify published studies; however, we may not have been able to identify all the synonyms for active learning classrooms. Our description of the active learning classrooms’ characteristics was based on that of Talbert and Mor-Avi (2019). However, Carlos et al., (2023) found additional designations for active learning spaces. We may also have missed studies due to our perceptions of health professional education. In addition, we did not search for gray literature, as we were only interested in peer-reviewed papers and aimed to provide an overview of published studies. It is the aim of the scoping review that determine whether gray literature should be included (Tricco et al., 2018). Our review also had some language limitations. Finally, the findings related to the thematic groups should be interpreted cautiously, as we, in line with the scoping review methodology, did not appraise the methodological quality of the included studies or synthesize the data. Due to the nature of the scoping review, design implications for policy and education need to be interpreted with caution.

## 6 Conclusion

The use of active learning classrooms in health professional education seems to facilitate interactions among students, between students and teachers, and between students and class content. Even though teachers’ and students’ freedom to move around and students’ common work surfaces can enhance a positive atmosphere and students’ engagement and active participation, it can also be challenging, especially due to the use of technology and the visibility of student groups’ work. Active learning classrooms’ features allow teachers to facilitate active learning. Both students and teachers may experience more active learning even when teachers use the same teaching approach in active learning classrooms as in traditional classrooms.

Our findings suggest that limited research has been conducted on the use of active learning classrooms in health professional education and that studies investigating active learning and in-class activities within the flipped classroom approach lack a description of the features of the room where the learning activities are offered. In addition, the didactics, teaching design, and interventions used should be better reported. Future research should explore how to facilitate health professional education teachers’ use of the opportunities in the active learning classrooms and students’ learning outcomes, as well as the effect of high-technology active learning classrooms versus low-technology active learning classrooms on the intended learning outcome. Currently, it seems that there are too few studies to conduct a qualitative or quantitative systematic review.

## Funding sources

No external funding.

## Declaration of competing interest

The authors declare that they have no known competing financial interests or personal relationships that could have appeared to influence the work reported in this paper.

## Appendix Supplementary materials

Image, application 1

Image, application 2

Image, application 3
