# Supplementary material for: Use of active learning classrooms in health professional education: A scoping review
Source: Int J Nurs Stud Adv. 2023 Nov 16;6:100167. doi: 10.1016/j.ijnsa.2023.100167 (PMC11080482; doi:10.1016/j.ijnsa.2023.100167)
Supplement: Supplementary file 1 [file mmc1.docx]

**Appendix 1. Changes in the protocol**

| **Protocol** | **Changes** |
| --- | --- |
| **Search**  Based on an initial search … build the search strategy in Ovid Medline using medical subject headings and text words. Then, the search strategy will be adopted to the other data bases. | **Search**  Based on an initial search … build the search strategy in Ovid Medline using text words to denote various types of active learning classrooms or similar environments. Medical subject headings were not used as no suitable terms were available to describe these environments. The strategy was piloted by the first and last authors. Then, the search strategy was adopted for the other databases. |
| **Selection of sources of evidence**  If there is uncertainty about whether a publication should be included, a third author will be consulted, and the final decision will be based on consensus. | **Selection of sources of evidence**  To ensure that the eligibility criteria were consistent across the pairs of reviewers, the authors discussed the meaning of the inclusion and exclusion criteria (Pollock et al., 2021).  When disagreement or uncertainty occurred, the pairs discussed whether a publication met the inclusion criteria. When the uncertainty about whether a publication should be included remained, the first author was consulted, and the final decision was based on consensus between the pair and the first author. |
| **Data charting process**  We will develop a standardized data charting form collecting the following information from the included papers; author, year, country; design; aim; sample (characteristics, sample size); use of technology in ALC (design attributes and digital and analogue tools); learning activities facilitated in ALC; didactics; and findings related to the research question.  Based on the piloting, the data charting form could be adjusted. Pairs of authors will extract data. One author will extract data, while the other will check accuracy. | **Data charting process**  We developed a standardized data charting form collecting the following information from the included papers: author, year, and country; design; aim; sample (characteristics, sample size); description of the active learning classroom, including use of technology in active learning classroom (design attributes and digital and analog tools), learning activities facilitated in active learning classroom, and didactics; and findings related to the research question.  Based on the piloting, the data charting form was adjusted. The pairs of authors extracted the data. One author extracted the data, whereas the other checked the accuracy. When a disagreement arose, the first and second authors independently extracted the data and made the final decision. |
