# Supplementary material for: Use of active learning classrooms in health professional education: A scoping review
Source: Int J Nurs Stud Adv. 2023 Nov 16;6:100167. doi: 10.1016/j.ijnsa.2023.100167 (PMC11080482; doi:10.1016/j.ijnsa.2023.100167)
Supplement: Supplementary file 2 [file mmc2.docx]

**Appendix 2: Search strategies**

| Database: Ovid MEDLINE(R) ALL | |
| --- | --- |
| # | **Query** |
| 1 | (active adj (learning or teaching or education or educational or instruction or instructional) adj2 (classroom* or environment or environments or center or centers or centre or centres or space or spaces)).tw,kf. |
| 2 | (AL adj2 classroom*).tw,kf. |
| 3 | (collaborative adj (learning or teaching or education or educational or instruction or instructional) adj2 (classroom* or environment or environments or center or centers or centre or centres or space or spaces)).tw,kf. |
| 4 | collaborative classroom*.tw,kf. |
| 5 | ((cooperative or co-operative) adj (learning or teaching or education or educational or instruction or instructional) adj2 (classroom* or environment or environments or center or centers or centre or centres or space or spaces)).tw,kf. |
| 6 | ((cooperative or co-operative) adj classroom*).tw,kf. |
| 7 | (CL adj2 classroom*).tw,kf. |
| 8 | (combined adj (learning or teaching or education or educational or instruction or instructional) adj2 (classroom* or environment or environments or center or centers or centre or centres or space or spaces)).tw,kf. |
| 9 | combined classroom*.tw,kf. |
| 10 | (innovative adj (learning or teaching or education or educational or instruction or instructional) adj2 (classroom* or environment or environments or center or centers or centre or centres or space or spaces)).tw,kf. |
| 11 | innovative classroom*.tw,kf. |
| 12 | ((interactive or "(inter)active" or inter-active) adj (learning or teaching or education or educational or instruction or instructional) adj2 (classroom* or environment or environments or center or centers or centre or centres or space or spaces)).tw,kf. |
| 13 | ((interactive or "(inter)active" or inter-active) adj classroom*).tw,kf. |
| 14 | (flexible adj (learning or teaching or education or educational or instruction or instructional) adj2 (classroom* or environment or environments or center or centers or centre or centres or space or spaces)).tw,kf. |
| 15 | flexible classroom*.tw,kf. |
| 16 | ("problem-based" adj (learning or teaching or education or educational or instruction or instructional) adj2 (classroom* or environment or environments or center or centers or centre or centres or space or spaces)).tw,kf. |
| 17 | problem-based classroom*.tw,kf. |
| 18 | (PBL adj2 (classroom* or environment or environments)).tw,kf. |
| 19 | (experiential adj (learning or teaching or education or educational or instruction or instructional) adj2 (classroom* or environment or environments or center or centers or centre or centres or space or spaces)).tw,kf. |
| 20 | experiential classroom*.tw,kf. |
| 21 | (EL adj2 classroom*).tw,kf. |
| 22 | ("technology-enhanced" adj3 (classroom* or environment or environments or center or centers or centre or centres or space or spaces)).tw,kf. |
| 23 | (TEL adj2 (classroom* or environment or environments)).tw,kf. |
| 24 | ("team-based" adj3 (classroom* or environment or environments or center or centers or centre or centres or space or spaces)).tw,kf. |
| 25 | (TBL adj2 (classroom* or environment or environments)).tw,kf. |
| 26 | ("technology-mediated" adj3 (classroom* or environment or environments or center or centers or centre or centres or space or spaces)).tw,kf. |
| 27 | (TML adj2 classroom*).tw,kf. |
| 28 | (("student-centered" or "student-centred") adj3 (classroom* or environment or environments or center or centers or centre or centres or space or spaces)).tw,kf. |
| 29 | (SCL adj2 classroom*).tw,kf. |
| 30 | (("learner-centered" or "learner-centred") adj3 (classroom* or environment or environments or center or centers or centre or centres or space or spaces)).tw,kf. |
| 31 | 1 or 2 or 3 or 4 or 5 or 6 or 7 or 8 or 9 or 10 or 11 or 12 or 13 or 14 or 15 or 16 or 17 or 18 or 19 or 20 or 21 or 22 or 23 or 24 or 25 or 26 or 27 or 28 or 29 or 30 |
| 32 | limit 31 to (yr="2012 -Current" and (danish or english or norwegian or portuguese or spanish or swedish)) |

| Database: Ovid Embase | |
| --- | --- |
| # | **Query** |
| 1 | (active adj (learning or teaching or education or educational or instruction or instructional) adj2 (classroom* or environment or environments or center or centers or centre or centres or space or spaces)).tw,kf. |
| 2 | (AL adj2 classroom*).tw,kf. |
| 3 | (collaborative adj (learning or teaching or education or educational or instruction or instructional) adj2 (classroom* or environment or environments or center or centers or centre or centres or space or spaces)).tw,kf. |
| 4 | collaborative classroom*.tw,kf. |
| 5 | ((cooperative or co-operative) adj (learning or teaching or education or educational or instruction or instructional) adj2 (classroom* or environment or environments or center or centers or centre or centres or space or spaces)).tw,kf. |
| 6 | ((cooperative or co-operative) adj classroom*).tw,kf. |
| 7 | (CL adj2 classroom*).tw,kf. |
| 8 | (combined adj (learning or teaching or education or educational or instruction or instructional) adj2 (classroom* or environment or environments or center or centers or centre or centres or space or spaces)).tw,kf. |
| 9 | combined classroom*.tw,kf. |
| 10 | (innovative adj (learning or teaching or education or educational or instruction or instructional) adj2 (classroom* or environment or environments or center or centers or centre or centres or space or spaces)).tw,kf. |
| 11 | innovative classroom*.tw,kf. |
| 12 | ((interactive or "(inter)active" or inter-active) adj (learning or teaching or education or educational or instruction or instructional) adj2 (classroom* or environment or environments or center or centers or centre or centres or space or spaces)).tw,kf. |
| 13 | ((interactive or "(inter)active" or inter-active) adj classroom*).tw,kf. |
| 14 | (flexible adj (learning or teaching or education or educational or instruction or instructional) adj2 (classroom* or environment or environments or center or centers or centre or centres or space or spaces)).tw,kf. |
| 15 | flexible classroom*.tw,kf. |
| 16 | ("problem-based" adj (learning or teaching or education or educational or instruction or instructional) adj2 (classroom* or environment or environments or center or centers or centre or centres or space or spaces)).tw,kf. |
| 17 | problem-based classroom*.tw,kf. |
| 18 | (PBL adj2 (classroom* or environment or environments)).tw,kf. |
| 19 | (experiential adj (learning or teaching or education or educational or instruction or instructional) adj2 (classroom* or environment or environments or center or centers or centre or centres or space or spaces)).tw,kf. |
| 20 | experiential classroom*.tw,kf. |
| 21 | (EL adj2 classroom*).tw,kf. |
| 22 | ("technology-enhanced" adj3 (classroom* or environment or environments or center or centers or centre or centres or space or spaces)).tw,kf. |
| 23 | (TEL adj2 (classroom* or environment or environments)).tw,kf. |
| 24 | ("team-based" adj3 (classroom* or environment or environments or center or centers or centre or centres or space or spaces)).tw,kf. |
| 25 | (TBL adj2 (classroom* or environment or environments)).tw,kf. |
| 26 | ("technology-mediated" adj3 (classroom* or environment or environments or center or centers or centre or centres or space or spaces)).tw,kf. |
| 27 | (TML adj2 classroom*).tw,kf. |
| 28 | (("student-centered" or "student-centred") adj3 (classroom* or environment or environments or center or centers or centre or centres or space or spaces)).tw,kf. |
| 29 | (SCL adj2 classroom*).tw,kf. |
| 30 | (("learner-centered" or "learner-centred") adj3 (classroom* or environment or environments or center or centers or centre or centres or space or spaces)).tw,kf. |
| 31 | 1 or 2 or 3 or 4 or 5 or 6 or 7 or 8 or 9 or 10 or 11 or 12 or 13 or 14 or 15 or 16 or 17 or 18 or 19 or 20 or 21 or 22 or 23 or 24 or 25 or 26 or 27 or 28 or 29 or 30 |
| 32 | limit 31 to (yr="2012 -Current" and (danish or english or norwegian or portuguese or spanish or swedish)) |

| Database: Ovid PsycINFO | |
| --- | --- |
| # | **Query** |
| 1 | collaborative learning/ |
| 2 | cooperative learning/ |
| 3 | experiential learning/ |
| 4 | problem based learning/ |
| 5 | 1 or 2 or 3 or 4 |
| 6 | classroom environment/ |
| 7 | 5 and 6 |
| 8 | (active adj (learning or teaching or education or educational or instruction or instructional) adj2 (classroom* or environment or environments or center or centers or centre or centres or space or spaces)).tw. |
| 9 | (AL adj2 classroom*).tw. |
| 10 | (collaborative adj (learning or teaching or education or educational or instruction or instructional) adj2 (classroom* or environment or environments or center or centers or centre or centres or space or spaces)).tw. |
| 11 | collaborative classroom*.tw. |
| 12 | ((cooperative or co-operative) adj (learning or teaching or education or educational or instruction or instructional) adj2 (classroom* or environment or environments or center or centers or centre or centres or space or spaces)).tw. |
| 13 | ((cooperative or co-operative) adj classroom*).tw. |
| 14 | (CL adj2 classroom*).tw. |
| 15 | (combined adj (learning or teaching or education or educational or instruction or instructional) adj2 (classroom* or environment or environments or center or centers or centre or centres or space or spaces)).tw. |
| 16 | combined classroom*.tw. |
| 17 | (innovative adj (learning or teaching or education or educational or instruction or instructional) adj2 (classroom* or environment or environments or center or centers or centre or centres or space or spaces)).tw. |
| 18 | innovative classroom*.tw. |
| 19 | ((interactive or "(inter)active" or inter-active) adj (learning or teaching or education or educational or instruction or instructional) adj2 (classroom* or environment or environments or center or centers or centre or centres or space or spaces)).tw. |
| 20 | ((interactive or "(inter)active" or inter-active) adj classroom*).tw. |
| 21 | (flexible adj (learning or teaching or education or educational or instruction or instructional) adj2 (classroom* or environment or environments or center or centers or centre or centres or space or spaces)).tw. |
| 22 | flexible classroom*.tw. |
| 23 | ("problem-based" adj (learning or teaching or education or educational or instruction or instructional) adj2 (classroom* or environment or environments or center or centers or centre or centres or space or spaces)).tw. |
| 24 | problem-based classroom*.tw. |
| 25 | (PBL adj2 (classroom* or environment or environments)).tw. |
| 26 | (experiential adj (learning or teaching or education or educational or instruction or instructional) adj2 (classroom* or environment or environments or center or centers or centre or centres or space or spaces)).tw. |
| 27 | experiential classroom*.tw. |
| 28 | (EL adj2 classroom*).tw. |
| 29 | ("technology-enhanced" adj3 (classroom* or environment or environments or center or centers or centre or centres or space or spaces)).tw. |
| 30 | (TEL adj2 (classroom* or environment or environments)).tw. |
| 31 | ("team-based" adj3 (classroom* or environment or environments or center or centers or centre or centres or space or spaces)).tw. |
| 32 | (TBL adj2 (classroom* or environment or environments)).tw. |
| 33 | ("technology-mediated" adj3 (classroom* or environment or environments or center or centers or centre or centres or space or spaces)).tw. |
| 34 | (TML adj2 classroom*).tw. |
| 35 | (("student-centered" or "student-centred") adj3 (classroom* or environment or environments or center or centers or centre or centres or space or spaces)).tw. |
| 36 | (SCL adj2 classroom*).tw. |
| 37 | (("learner-centered" or "learner-centred") adj3 (classroom* or environment or environments or center or centers or centre or centres or space or spaces)).tw. |
| 38 | 7 or 8 or 9 or 10 or 11 or 12 or 13 or 14 or 15 or 16 or 17 or 18 or 19 or 20 or 21 or 22 or 23 or 24 or 25 or 26 or 27 or 28 or 29 or 30 or 31 or 32 or 33 or 34 or 35 or 36 or 37 |
| 39 | limit 38 to (yr="2012 -Current" and (danish or english or norwegian or portuguese or spanish or swedish)) |

| Database: CINAHL Complete via EBSCO Host | | |
| --- | --- | --- |
| # | **Query** | **Limiters/Expanders** |
| S1 | TI(active W0 (learning or teaching or education or educational or instruction or instructional) N1 (classroom* or environment or environments or center or centers or centre or centres or space or spaces)) OR AB(active W0 (learning or teaching or education or educational or instruction or instructional) N1 (classroom* or environment or environments or center or centers or centre or centres or space or spaces)) | Search modes - Boolean/Phrase |
| S2 | TI(AL N1 classroom*) OR AB(AL N1 classroom*) | Search modes - Boolean/Phrase |
| S3 | TI(collaborative W0 (learning or teaching or education or educational or instruction or instructional) N1 (classroom* or environment or environments or center or centers or centre or centres or space or spaces)) OR AB(collaborative W0 (learning or teaching or education or educational or instruction or instructional) N1 (classroom* or environment or environments or center or centers or centre or centres or space or spaces)) | Search modes - Boolean/Phrase |
| S4 | TI("collaborative classroom*") OR AB("collaborative classroom*") | Search modes - Boolean/Phrase |
| S5 | TI((cooperative OR co-operative) W0 (learning or teaching or education or educational or instruction or instructional) N1 (classroom* or environment or environments or center or centers or centre or centres or space or spaces)) OR AB((cooperative OR co-operative) W0 (learning or teaching or education or educational or instruction or instructional) N1 (classroom* or environment or environments or center or centers or centre or centres or space or spaces)) | Search modes - Boolean/Phrase |
| S6 | TI("cooperative classroom*" OR "co-operative classroom*") OR AB("cooperative classroom*" OR "co-operative classroom*") | Search modes - Boolean/Phrase |
| S7 | TI(CL N1 classroom*) OR AB(CL N1 classroom*) | Search modes - Boolean/Phrase |
| S8 | TI(combined W0 (learning or teaching or education or educational or instruction or instructional) N1 (classroom* or environment or environments or center or centers or centre or centres or space or spaces)) OR AB(combined W0 (learning or teaching or education or educational or instruction or instructional) N1 (classroom* or environment or environments or center or centers or centre or centres or space or spaces)) | Search modes - Boolean/Phrase |
| S9 | TI("combined classroom*") OR AB("combined classroom*") | Search modes - Boolean/Phrase |
| S10 | TI(innovative W0 (learning or teaching or education or educational or instruction or instructional) N1 (classroom* or environment or environments or center or centers or centre or centres or space or spaces)) OR AB(innovative W0 (learning or teaching or education or educational or instruction or instructional) N1 (classroom* or environment or environments or center or centers or centre or centres or space or spaces)) | Search modes - Boolean/Phrase |
| S11 | TI("innovative classroom*") OR AB("innovative classroom*") | Search modes - Boolean/Phrase |
| S12 | TI((interactive OR "(inter)active" OR inter-active) W0 (learning or teaching or education or educational or instruction or instructional) N1 (classroom* or environment or environments or center or centers or centre or centres or space or spaces)) OR AB((interactive OR "(inter)active" OR inter-active) W0 (learning or teaching or education or educational or instruction or instructional) N1 (classroom* or environment or environments or center or centers or centre or centres or space or spaces)) | Search modes - Boolean/Phrase |
| S13 | TI("interactive classroom*" OR "(inter)active classroom*" OR "inter-active classroom*") OR AB("interactive classroom*" OR "(inter)active classroom*" OR "inter-active classroom*") | Search modes - Boolean/Phrase |
| S14 | TI(flexible W0 (learning or teaching or education or educational or instruction or instructional) N1 (classroom* or environment or environments or center or centers or centre or centres or space or spaces)) OR AB(flexible W0 (learning or teaching or education or educational or instruction or instructional) N1 (classroom* or environment or environments or center or centers or centre or centres or space or spaces)) | Search modes - Boolean/Phrase |
| S15 | TI("flexible classroom*") OR AB("flexible classroom*") | Search modes - Boolean/Phrase |
| S16 | TI("problem-based" W0 (learning or teaching or education or educational or instruction or instructional) N1 (classroom* or environment or environments or center or centers or centre or centres or space or spaces)) OR AB("problem-based" W0 (learning or teaching or education or educational or instruction or instructional) N1 (classroom* or environment or environments or center or centers or centre or centres or space or spaces)) | Search modes - Boolean/Phrase |
| S17 | TI("problem-based classroom*") OR AB("problem-based classroom*") | Search modes - Boolean/Phrase |
| S18 | TI(PBL N1 (classroom* or environment or environments)) OR AB(PBL N1 (classroom* or environment or environments)) | Search modes - Boolean/Phrase |
| S19 | TI(experiential W0 (learning or teaching or education or educational or instruction or instructional) N1 (classroom* or environment or environments or center or centers or centre or centres or space or spaces)) OR AB(experiential W0 (learning or teaching or education or educational or instruction or instructional) N1 (classroom* or environment or environments or center or centers or centre or centres or space or spaces)) | Search modes - Boolean/Phrase |
| S20 | TI("experiential classroom*") OR AB("experiential classroom*") | Search modes - Boolean/Phrase |
| S21 | TI("EL" N1 classroom*) OR AB("EL" N1 classroom*) | Search modes - Boolean/Phrase |
| S22 | TI("technology-enhanced" N2 (classroom* or environment or environments or center or centers or centre or centres or space or spaces)) OR AB("technology-enhanced" N2 (classroom* or environment or environments or center or centers or centre or centres or space or spaces)) | Search modes - Boolean/Phrase |
| S23 | TI(TEL N1 (classroom* or environment or environments)) OR AB(TEL N1 (classroom* or environment or environments)) | Search modes - Boolean/Phrase |
| S24 | TI("team-based" N2 (classroom* or environment or environments or center or centers or centre or centres or space or spaces)) OR AB("team-based" N2 (classroom* or environment or environments or center or centers or centre or centres or space or spaces)) | Search modes - Boolean/Phrase |
| S25 | TI(TBL N1 (classroom* or environment or environments)) OR AB(TBL N1 (classroom* or environment or environments)) | Search modes - Boolean/Phrase |
| S26 | TI("technology-mediated" N2 (classroom* or environment or environments or center or centers or centre or centres or space or spaces)) OR AB("technology-mediated" N2 (classroom* or environment or environments or center or centers or centre or centres or space or spaces)) | Search modes - Boolean/Phrase |
| S27 | TI(TML N1 classroom*) OR AB(TML N1 classroom*) | Search modes - Boolean/Phrase |
| S28 | TI(("student-centered" or "student-centred") N2 (classroom* or environment or environments or center or centers or centre or centres or space or spaces)) OR AB(("student-centered" or "student-centred") N2 (classroom* or environment or environments or center or centers or centre or centres or space or spaces)) | Search modes - Boolean/Phrase |
| S29 | TI(SCL N1 classroom*) OR AB(SCL N1 classroom*) | Search modes - Boolean/Phrase |
| S30 | TI(("learner-centered" or "learner-centred") N2 (classroom* or environment or environments or center or centers or centre or centres or space or spaces)) OR AB(("learner-centered" or "learner-centred") N2 (classroom* or environment or environments or center or centers or centre or centres or space or spaces)) | Search modes - Boolean/Phrase |
| S31 | S1 OR S2 OR S3 OR S4 OR S5 OR S6 OR S7 OR S8 OR S9 OR S10 OR S11 OR S12 OR S13 OR S14 OR S15 OR S16 OR S17 OR S18 OR S19 OR S20 OR S21 OR S22 OR S23 OR S24 OR S25 OR S26 OR S27 OR S28 OR S29 OR S30 | Search modes - Boolean/Phrase |
| S32 | S1 OR S2 OR S3 OR S4 OR S5 OR S6 OR S7 OR S8 OR S9 OR S10 OR S11 OR S12 OR S13 OR S14 OR S15 OR S16 OR S17 OR S18 OR S19 OR S20 OR S21 OR S22 OR S23 OR S24 OR S25 OR S26 OR S27 OR S28 OR S29 OR S30 | Limiters - Published Date: 20120101-20221231; Language: Danish, English, Norwegian, Portuguese, Spanish, Swedish |

| Database: ERIC via EBSCO Host | | |
| --- | --- | --- |
| # | **Query** | **Limiters/Expanders** |
| S1 | ((DE "Active Learning") OR (DE "Experiential Learning") OR (DE "Problem Based Learning") OR (DE "Cooperative Learning")) AND ((DE "Classroom Environment") OR (DE "Classroom Design")) | Search modes - Boolean/Phrase |
| S2 | TI(active W0 (learning or teaching or education or educational or instruction or instructional) N1 (classroom* or environment or environments or center or centers or centre or centres or space or spaces)) OR AB(active W0 (learning or teaching or education or educational or instruction or instructional) N1 (classroom* or environment or environments or center or centers or centre or centres or space or spaces)) | Search modes - Boolean/Phrase |
| S3 | TI(AL N1 classroom*) OR AB(AL N1 classroom*) | Search modes - Boolean/Phrase |
| S4 | TI(collaborative W0 (learning or teaching or education or educational or instruction or instructional) N1 (classroom* or environment or environments or center or centers or centre or centres or space or spaces)) OR AB(collaborative W0 (learning or teaching or education or educational or instruction or instructional) N1 (classroom* or environment or environments or center or centers or centre or centres or space or spaces)) | Search modes - Boolean/Phrase |
| S5 | TI("collaborative classroom*") OR AB("collaborative classroom*") | Search modes - Boolean/Phrase |
| S6 | TI((cooperative OR co-operative) W0 (learning or teaching or education or educational or instruction or instructional) N1 (classroom* or environment or environments or center or centers or centre or centres or space or spaces)) OR AB((cooperative OR co-operative) W0 (learning or teaching or education or educational or instruction or instructional) N1 (classroom* or environment or environments or center or centers or centre or centres or space or spaces)) | Search modes - Boolean/Phrase |
| S7 | TI("cooperative classroom*" OR "co-operative classroom*") OR AB("cooperative classroom*" OR "co-operative classroom*") | Search modes - Boolean/Phrase |
| S8 | TI(CL N1 classroom*) OR AB(CL N1 classroom*) | Search modes - Boolean/Phrase |
| S9 | TI(combined W0 (learning or teaching or education or educational or instruction or instructional) N1 (classroom* or environment or environments or center or centers or centre or centres or space or spaces)) OR AB(combined W0 (learning or teaching or education or educational or instruction or instructional) N1 (classroom* or environment or environments or center or centers or centre or centres or space or spaces)) | Search modes - Boolean/Phrase |
| S10 | TI("combined classroom*") OR AB("combined classroom*") | Search modes - Boolean/Phrase |
| S11 | TI(innovative W0 (learning or teaching or education or educational or instruction or instructional) N1 (classroom* or environment or environments or center or centers or centre or centres or space or spaces)) OR AB(innovative W0 (learning or teaching or education or educational or instruction or instructional) N1 (classroom* or environment or environments or center or centers or centre or centres or space or spaces)) | Search modes - Boolean/Phrase |
| S12 | TI("innovative classroom*") OR AB("innovative classroom*") | Search modes - Boolean/Phrase |
| S13 | TI((interactive OR "(inter)active" OR inter-active) W0 (learning or teaching or education or educational or instruction or instructional) N1 (classroom* or environment or environments or center or centers or centre or centres or space or spaces)) OR AB((interactive OR "(inter)active" OR inter-active) W0 (learning or teaching or education or educational or instruction or instructional) N1 (classroom* or environment or environments or center or centers or centre or centres or space or spaces)) | Search modes - Boolean/Phrase |
| S14 | TI("interactive classroom*" OR "(inter)active classroom*" OR "inter-active classroom*") OR AB("interactive classroom*" OR "(inter)active classroom*" OR "inter-active classroom*") | Search modes - Boolean/Phrase |
| S15 | TI(flexible W0 (learning or teaching or education or educational or instruction or instructional) N1 (classroom* or environment or environments or center or centers or centre or centres or space or spaces)) OR AB(flexible W0 (learning or teaching or education or educational or instruction or instructional) N1 (classroom* or environment or environments or center or centers or centre or centres or space or spaces)) | Search modes - Boolean/Phrase |
| S16 | TI("flexible classroom*") OR AB("flexible classroom*") | Search modes - Boolean/Phrase |
| S17 | TI("problem-based" W0 (learning or teaching or education or educational or instruction or instructional) N1 (classroom* or environment or environments or center or centers or centre or centres or space or spaces)) OR AB("problem-based" W0 (learning or teaching or education or educational or instruction or instructional) N1 (classroom* or environment or environments or center or centers or centre or centres or space or spaces)) | Search modes - Boolean/Phrase |
| S18 | TI("problem-based classroom*") OR AB("problem-based classroom*") | Search modes - Boolean/Phrase |
| S19 | TI(PBL N1 (classroom* or environment or environments)) OR AB(PBL N1 (classroom* or environment or environments)) | Search modes - Boolean/Phrase |
| S20 | TI(experiential W0 (learning or teaching or education or educational or instruction or instructional) N1 (classroom* or environment or environments or center or centers or centre or centres or space or spaces)) OR AB(experiential W0 (learning or teaching or education or educational or instruction or instructional) N1 (classroom* or environment or environments or center or centers or centre or centres or space or spaces)) | Search modes - Boolean/Phrase |
| S21 | TI("experiential classroom*") OR AB("experiential classroom*") | Search modes - Boolean/Phrase |
| S22 | TI("EL" N1 classroom*) OR AB("EL" N1 classroom*) | Search modes - Boolean/Phrase |
| S23 | TI("technology-enhanced" N2 (classroom* or environment or environments or center or centers or centre or centres or space or spaces)) OR AB("technology-enhanced" N2 (classroom* or environment or environments or center or centers or centre or centres or space or spaces)) | Search modes - Boolean/Phrase |
| S24 | TI(TEL N1 (classroom* or environment or environments)) OR AB(TEL N1 (classroom* or environment or environments)) | Search modes - Boolean/Phrase |
| S25 | TI("team-based" N2 (classroom* or environment or environments or center or centers or centre or centres or space or spaces)) OR AB("team-based" N2 (classroom* or environment or environments or center or centers or centre or centres or space or spaces)) | Search modes - Boolean/Phrase |
| S26 | TI(TBL N1 (classroom* or environment or environments)) OR AB(TBL N1 (classroom* or environment or environments)) | Search modes - Boolean/Phrase |
| S27 | TI("technology-mediated" N2 (classroom* or environment or environments or center or centers or centre or centres or space or spaces)) OR AB("technology-mediated" N2 (classroom* or environment or environments or center or centers or centre or centres or space or spaces)) | Search modes - Boolean/Phrase |
| S28 | TI(TML N1 classroom*) OR AB(TML N1 classroom*) | Search modes - Boolean/Phrase |
| S29 | TI(("student-centered" or "student-centred") N2 (classroom* or environment or environments or center or centers or centre or centres or space or spaces)) OR AB(("student-centered" or "student-centred") N2 (classroom* or environment or environments or center or centers or centre or centres or space or spaces)) | Search modes - Boolean/Phrase |
| S30 | TI(SCL N1 classroom*) OR AB(SCL N1 classroom*) | Search modes - Boolean/Phrase |
| S31 | TI(("learner-centered" or "learner-centred") N2 (classroom* or environment or environments or center or centers or centre or centres or space or spaces)) OR AB(("learner-centered" or "learner-centred") N2 (classroom* or environment or environments or center or centers or centre or centres or space or spaces)) | Search modes - Boolean/Phrase |
| S32 | S1 OR S2 OR S3 OR S4 OR S5 OR S6 OR S7 OR S8 OR S9 OR S10 OR S11 OR S12 OR S13 OR S14 OR S15 OR S16 OR S17 OR S18 OR S19 OR S20 OR S21 OR S22 OR S23 OR S24 OR S25 OR S26 OR S27 OR S28 OR S29 OR S30 OR S31 | Search modes - Boolean/Phrase |
| S33 | S1 OR S2 OR S3 OR S4 OR S5 OR S6 OR S7 OR S8 OR S9 OR S10 OR S11 OR S12 OR S13 OR S14 OR S15 OR S16 OR S17 OR S18 OR S19 OR S20 OR S21 OR S22 OR S23 OR S24 OR S25 OR S26 OR S27 OR S28 OR S29 OR S30 OR S31 | Limiters - Date Published: 20120101-20221231; Language: Danish, English, Portuguese, Spanish; Castilian, Swedish |
