# Supplementary material for: Use of active learning classrooms in health professional education: A scoping review
Source: Int J Nurs Stud Adv. 2023 Nov 16;6:100167. doi: 10.1016/j.ijnsa.2023.100167 (PMC11080482; doi:10.1016/j.ijnsa.2023.100167)
Supplement: Supplementary file 3 [file mmc3.docx]

**Appendix 3. Characteristics of the included studies**

| **Author, year, country** | **Design** | **Aim** | **Sample** | **Description of the ALC** | **Learning activities facilitated in the ALC.**  **Didactics.** | **Results** |
| --- | --- | --- | --- | --- | --- | --- |
| Basdogan and Morrone (2021)  US | Mixed methods | To focus on the behaviors of the actors (i.e., faculty and students) in an active learning classroom (ALC), the Collaboration Café, to capture, interpret, and report their use of technology, space, and pedagogy over an eight‐week period | Public health teacher (*n*=1), gender: male, age: not reported (NR) | Collaboration Café used at School of Public Health in Recreational Therapy Course  Equipped with:   - Movable chairs and tables, sofa seating with small coffee tables - Projector and screen, computer at the station, whiteboard for teacher use - Laptops and portable whiteboards for student use - Clickers - Room’s capacity is 49 | Learning activities: PowerPoint presentations and videos  Interactive dialogs and the use of clickers  Didactic: Direct instruction, monitoring, and consulting | Direct instruction and monitoring were the most prevalent pedagogical behavior used by the public health teacher, followed by consultations and interactive dialogs. The projector was the most used technology to show PowerPoint presentations and videos. The teacher also used clickers during presentations, which decreased the time spent using the computer. The second most used technology was the students’ laptops. The teacher spent half of his time roaming in the room and half of his time standing in specific locations, including the teacher´s computer, small groups, and the middle area |
| Beery et al. (2013)  US | Mixed methods | To evaluate teaching behaviors in traditional and collaborative classrooms | Nursing teachers (*n*=4), gender: NR, age: NR | The collaborative classroom at a university’s college of nursing.  Equipped with:   - Mobile chairs - Each group table: a 60-inch LCD screen, dedicated networked computer, webcam, wireless keyboard and mouse, tablet, and dry-erase marker set - Color-coded group icons - Synchronous collaboration software - Duplicated primary displays - Mobile hanging whiteboards for student and teacher use - Copy camera to digitally capture whiteboards - Opportunity for students and teachers to retrieve digital copies from an internal website | Learning activities: Lectures with PowerPoint presentations,  questions, explanations, and  discussions  Didactic: NR | No significant difference between teachers in the collaborative classroom and teachers in the traditional classroom regarding the use of active learning pedagogies, passive learning, and teacher interactions with students |
| Bruner et al., 2022  US | Quasi- experimental design | To contribute to research on active learning by addressing the problem of disentangling the effects of classroom architecture, student characteristics, and pedagogical design as they relate to student achievement | Master’s degree students in speech–language pathology, 1. year (*n*=79) (Traditional classrooms [TC] *n*=39, ALC *n*=40), majority females, age: NR | The ALC. Equipped with:   - Tables for 6–8 students - Each table has an LCD screen that could be connected to the student’s laptop and a wall-mounted whiteboard nearby - Microphones and acoustic amplification for students and teachers | Learning activities: Individual quizzes.  Teams of students, three problem-based learning assignments require students to identify and solve problems and apply foundational knowledge  Didactic: Flipped classroom (FC) | Students score the ALC significantly higher than the TC, special regarding feelings about the classroom.  Neither student perceptions of the classroom spaces nor the spaces themselves impacted course grades; however, the pedagogy employed by the teacher and student course experiences did; experiences are positively related to working with peers, and pedagogy is positively related to teaching and learning with peers and negatively related to displaying student work in front of the class |
| Donkin and Kynn (2021)  Australia | Mixed methods | To evaluate the impact of a technology-enabled collaboration studio on facilitating team-based learning (TBL) using explicit assessment items as objective student learning outcomes and implicit subjective, self-reported feelings of engagement and readiness for clinical practice | Undergraduate biomedicine students in hematology, 2 years (*n*=105), (TC *n*=44, ALC *n*=61), majority females, majority aged 20 years or older | The collaboration studio at a regional university  Equipped with:   - 10 group stations for 3–6 students - Each group desktop touchscreen computers and Wi-Fi, linked to a multi-source ultra-wide lectern screen - Work within or across groups—using the central display monitor that projected onto the lectern screen, configured to a single display or as many as 10 displays - All students could view and comment on the central display containing each group’s activities - Room’s capacity is 60 | Learning activities: Feedback (simulated clinical case studies), assessment of individual (case study examination), and peer review through group case study presentations  Didactic: TBL | Students in the collaboration studio performed worse on the group presentation, better on individual practical case study examination, and statistically better on the final exam than students in the traditional classroom. Students found technology in the ALC useful and enjoyable, whereas some felt that the technology wasted time. The ALC improved the group work experience and more interactions, and the technology was applied to work-related scenarios. The students interacted with real-life examples. Students found the ALC environment more exciting and increased motivation, and they received more attention from the teachers. |
| Gordy et al. (2018)  US | Qualitative interpretive design | To investigate the impact of ALC on teaching and learning | Dental teacher teaching dental radiology (*n*=1), gender: NR, age: NR  Focus group, teachers from health courses (*n*=8), gender: NR, age: NR  Dental students in radiology (*n*=275), gender: NR, age: NR  Survey, students (*n*=193 out of 275), gender: NR, age: NR | The Collaboratory at an Academic Medical Center  equipped with:   - 12 large interconnected flat screens and a wall projector - Semicircular and rectangular tables with access to power outlets and smart device hook-ups for personal devices - Swivel chairs with built-in work surfaces and storage for personal belongings - Large and small portable whiteboards | Learning activities: Group work and Individual work  Group presentations and discussions  Role play  Peer teaching  Didactic: NR | The non-hierarchical design of the ALC and the opportunity to move around democratized learning and enhance classroom interactions (both human-to-human and human-to-non-human interactions). The physical features of the ALC created a positive environment for both students and teachers, feeling welcomed and relaxed. The ALC provided students with a more effective workspace for group activities than the TC, and the environment fostered the development of creativity and allowed students to be involved in higher-order thinking. The design of the ALC allowed mobility, promoted student engagement, and students appeared to stay more engaged than in the TC |
| Gordy et al. (2019)  US | Mixed methods | To discover how an ALC influenced the teaching and learning of dental radiology with dental hygiene students | Dental hygiene teacher teaching dental radiology (*n*=1), gender: NR, age: NR  Dental hygiene students in dental radiology, 1 year (*n*=19) and 2 years (*n*=19).  Survey, students (*n*=32 out of 38), majority females, age: NR | The Collaboratory at the University Medical Center  Equipped with:   - Multiple large interconnected flat-panel screens on the walls and at the tables - Each table has power outlets and smart device hook-ups for personal devices - Swivel chairs with built-in work surfaces and storage for personal belongings - Portable whiteboards on rolling stands placed in the corners of the room | Learning activities: Group work and discussion. Individual activities follow by share, discuss, and present.  Teacher shifts from one mode of teaching to another offering different classroom activities  Didactic: NR | Students and teachers enjoyed the ALC’s comfort and spaciousness, and the technology facilitated viewing instructional content, information sharing, and classroom engagement. Most students preferred to take classes in the ALC rather than in the TC. The teacher devoted more time to discussion or group work in the ALC, and the students experienced more interactions with the teacher, peers, and the material being taught. The ALC provided adequate space for group work and helped enhance the efficiency of group work and information sharing. The teacher experienced challenges, such as the inability to make visual contact with students, and some students experienced distractions in the ALC and preferred TC |
| Gordy et al. (2020)  US | Mixed methods | To find out what interactions are taking place in the active learning spaces and how they affect student learning | Focus groups, students: (*n*=38) (physical therapy [*n*=10]; occupational therapy, [*n*=12]; dental hygiene, [*n*=11]; biostatistics, and data science, [*n*=5]), majority females, majority aged between 21 and 25 years  Survey, students (*n*=177 out of 204, *n*=175 included), gender: NR, age: NR | The Collaboratory at the University of Mississippi Medical Center  Equipped with:   - Multiple large interconnected flat-panel screens on the walls and at the tables - Each table has power outlets and smart device hook-ups for personal devices - Swivel chairs with built-in work surfaces and storage for personal belongings - Writeable walls or portable whiteboards on rolling stands placed in the corners of the room | Learning activities: Group work (case studies, hands-on)  Didactic: NR | Spatial equity and a non-threatening atmosphere put students in positive mindsets and triggered active participation. The screen enabled the visual convenience of learning and engaged students. Screen sharing allowed for effective collaboration with peers. The group work conducted in the ALC facilitated mutual learning and fostered idea generation. Peer collaboration and interaction in the ALC promoted higher extrinsic motivation and learning accountability and reduced the occurrence of social loafing. The environment broke the norm of homophily and improved reciprocated peer relations. Increased interactions with heterogeneous groups of peers promoted overall peer relations in class. These results on group dynamics were reinforced when comparing the ALC with TC |
| Lee et al. (2018)  US | Mixed methods | To investigate how the spatial and technological features of a large collaborative classroom support active learning based on the Pedagogy-Space-Technology framework | Survey, public health students, 2 years (*n*=17), and 3 years (*n*=15), gender: NR, age: NR | The Collaborative Learning Studio at a University  Equipped with:   - 16 U-shaped tables for 6 students - Each student table contains a desktop, a wall-mounted LED monitor, connections for laptops, a document camera, and a portable whiteboard - Teacher’s stations in the front corner and the center, video wall, control panels, wall-mounted projectors/screens, speaker and push-to-talk microphones at student tables, teacher´s desktop and controls, and wireless microphone - Two levels: lower and upper classrooms - Room’s capacity is 96 | Learning activities: Lectures, one way or interactive before or after group activities. Group activities, group discussions, and class-wide discussions.  Individual instructional activity.  Movie or other audio-visual presentations  Didactic: NR | The students’ computers were the most frequently used technology for group work, and student desktops were used at almost every class meeting. The video wall for presenting student work was used for facilitating class-wide discussion |
| Marchiori and McLean (2022)  Canada | Experimental design | To investigate the impact of the ALC on the students’ perception of the development of effective communication and to investigate whether the ALC affects the quality of peer-to-peer interactions | Undergraduate medical students, 4 years (*n*=33), males (*n*=19), females (*n*=14), aged mean average 21.42 ± 1.62 years. Not all students are present during data collection, *n* value changed | ALC at School of Medicine  Equipped with:   - Round tables for students - Each group table has its own projector screen, interactive whiteboard - Teacher located in the center of the room with groups of students around the periphery of the room | Learning activities: Before coming to class, complete online learning modules; during class, different active learning activities designed to build upon students’ baseline knowledge acquired from the modules (think-pair-share, working on a case study, defining terms with a group, and different forms of group discussion)  Didactic: FC | Students behave similarly in the ALC and TC, performing in-class activities.  Students preferred the ALC for supporting communication skill development due to group work/collaboration and student–student interactions, despite it having no quantifiable effect on their communication apprehension.  Students experienced that ALC had a significantly higher impact than the TC on group work and collaboration and student-student interactions, but statements indicated different experiences regarding physical space and technology |
| Metzger et al. (2020) US | Cross-sectional design | To describe the variety of active engagements that characterize students’ behaviors in ALC | Undergraduate health sciences students, *n* NR, gender: NR, age: NR | Classroom design and use of tools: NR | Learning activities: NR  Didactic: NR | Out of 10 possible engagement behaviors, eight were observed in the ALC, and two were never observed (Creating/Constructing and Performing/Presenting).  The number of engagements was an average of 4.9 across upper and lower-division courses and STEM and non-STEM courses. The engagement profile between the early and late observations was occasionally consistent.  Three engagement behaviors (listening/processing, discussing, and problem solving) accounted for approximately all the student time observed. Most frequently listening/processing, but the majority of student engagement observed was in other engagement types (discussing, problem solving, writing/drawing, reading) |
| Seet et al. (2022)  Singapore | Quasi-experimental design | To investigate the effects of seating distance from the tutor and orientation within the team on engagement in novice and experienced learners in classrooms designed for TBL | Medical students, 1 year (*n*=85) and 2 years (*n*=75),  majority males aged 18 and 23 years, mean age 19.5 years | Classroom at School of Medicine  Equipped with:   - Groups of 5–7 - Dual-tiered seating circular layout - 6 big screens all around the periphery of the room - Chairs with wheels - Fixed tables - Microphones for each group - Teacher in front | Learning activities: Readiness assurances (closed book multiple choice test individual and in the team, follow by class discussion and teacher´s clarification.  Application exercise (open book, teamwork, and intrateam discussions, followed by inter-team discussion, and teacher´s explanation to the whole class)  Didactic: TBL | Regarding seating distance to the teacher, the seating of second-year students did not significantly affect their engagement. First-year students exhibited a stronger preference to sit nearer the teacher, and their engagement levels decreased significantly for those who moved further. Overall engagement decreased post-swap, regardless of the direction moved. For first-year and second-year students, seating orientation did not significantly affect engagement, but both groups preferred sitting with their front facing the teacher |
